# Dynamic Response and Deformative Mechanism of the Shape Memory Polymer Filled with Low-Melting-Point Alloy under Different Dynamic Loads

**DOI:** 10.3390/polym15020423

**Published:** 2023-01-13

**Authors:** Huanhuan Wang, Yongqiang Zhang, Zhuhua Tan

**Affiliations:** 1 School of Mechanical Engineering, Hebei University of Technology, Tianjin 300104, China; 2 Institute of Fluid Physics, China Academy of Engineering Physics, Mianyang 621900, China

**Keywords:** shape memory polymer, low-melting-point alloy, dynamic mechanical properties, high strain rate, deformation mechanism

## Abstract

Low-melting-point alloy (LMPA) was used as an additive to prepare epoxy-resin-based shape memory polymer composites (LMPA/EP SMP), and dynamic mechanical analyzer (DMA) tests were performed to demonstrate the shape memory effect, storage modulus, and stiffness of the composites under different load cases. The composites exhibited an excellent shape recovery ratio and shape fixity ratio, and a typical turning point was observed in the storage modulus curves, which was attributed to the melting of the LMPA. In order to investigate the dynamic deformation mechanism at high strain rates, split Hopkinson pressure bar (SHPB) experiments were performed to study the influence of the strain rate and plastic work on the dynamic mechanical response of LMPA/EP composites. The results showed that there was a saturated tendency for the flow stress with increasing strain rate, and the composites exhibited a typical brittle failure mode at high strain rate. Moreover, an obvious melting phenomenon of the LMPA was observed by SEM tests, which was due to the heat generated by the plastic work at high strain rate. The fundamental of the paper provided an effective approach to modulate the stiffness and evaluate the characteristics of SMP composites.

## 1. Introduction

Shape memory polymers (SMPs) are a class of smart materials that has the ability to maintain a temporary configuration and recover to the original shape under external stimuli, such as heat [1,2], light [3], electric [4,5], and magnetic field [6]. SMPs have promising applications in the field of aerospace [7,8], medical [9,10], military [11], and civil engineering [12,13] due to their low density, special shape recovery ratio, and excellent mechanical properties [14,15]. Due to being convenient to control the temperature, the heat-induced shape memory materials have attracted lots of interest. Lots of work has been performed on the design and fabrication of the heat-induced SMPs, and different types of heat-induced SMPs materials have been developed, including one-way [16,17,18], two-way [19,20,21], and multiple [21,22] SMPs materials.

With the increasing requirement of variable stiffness in the field of robots grasping, medical devices, and aerospace equipment, different additives have been introduced into SMP materials to achieve the variable stiffness performance [15,23,24,25,26,27,28,29,30,31,32,33,34,35], including Field’s metal [15], NiTi alloy [26], and polycaprolactone (PCL) [27]. Though different approaches have been adopted to achieve tunable stiffness, it is still a challenge to develop new materials to achieve variable stiffness performance. Low-melting-point alloy (LMPA) is an excellent candidate to design the composites with variable stiffness due to its low melting point of 46 °C [15], good contribution to the shape fixation ratio [28,29,30], and tunable stiffness [15,25,32,33,34,35]. Thus, it is worth developing new composites with both shape memory ability and tunable stiffness performance by introducing LMPA into an epoxy resin matrix.

Moreover, many works have been performed on the shape memory performance of SMP [1,2,18,27]. However, some applications of SMP involve the impact and shock loads during the service life, such as aerospace structures subjected to shock loading in the period of service on orbit. There is a significant difference in the mechanical properties between quasi-static and impact loading. Thus, more attention should be paid to the dynamic mechanical properties of the SMPs. Fortunately, some work has been performed on the dynamic mechanical performance of the polymers at high strain rates, which paves the way to explore the mechanical performance of the SMPs under shock loading. Lei et al. [36] found that polyvinyl chloride elastomers exhibit super-elastic and viscoelastic properties under static and dynamic loads, respectively. Fan et al. [37] found that the impact load would cause a significant temperature rise of polyurethane elastomer materials. Tamrakar et al. [38] recognized that the thermal softening of DER353 epoxies became more pronounced with increasing strain rates. Bakbak et al. [39] found that the elastic modulus and yield stress of LY564 epoxy resin increased significantly with the increase in strain rate. Minh et al. [40] studied the fatigue performance of polypropylene (PP) at various amplitudes and frequencies on fatigue cycles under tensile test conditions. Sassi et al. [41] investigated the effects of the strain rate on the dynamic compressive response and the failure behavior of polyester matrix.

Based on the previous work on SMP with variable stiffness, the low-point-melting alloy (LMPA) was introduced into an epoxy resin matrix to fabricate SMP materials with variable stiffness, and the corresponding DMA experiments were carried out to demonstrate the storage modulus, variable stiffness, and shape memory effect of the LMPA/EP composites. Moreover, in order to support the application of LMPA/EP composites in the environment of impact loading, the dynamic mechanical properties were investigated by using the split Hopkinson pressure bar, and the corresponding deformation mechanism at high strain rates was also discussed in this paper.

## 2. Experimental Section

### 2.1. Materials

**Figure 1:**
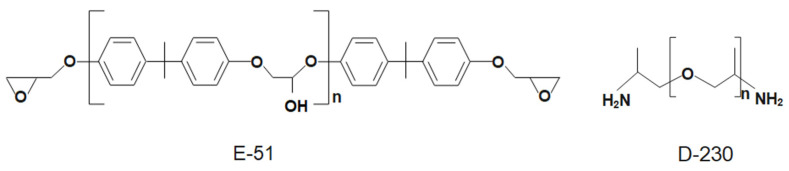
The chemical structural formula of E-51 and D-230.

The bisphenol-A epoxy resin, E-51, was purchased from Shanghai Aotun Chemical Technology Co., Ltd. Shanghai, China. The curing agent, D-230, which is a colorless liquid and primary amine (PA) accounting for more than 97% of the total amine value, was purchased from Shanghai McLean Biochemical Technology Co., Ltd., Shanghai, China. The chemical structural formulae of E-51 and D-230 are shown in Figure 1. Filler, which is a lead-based low-melting-point alloy that melts at 47 °C with the following percentages by weight: Bi 49.2%, Pb 22.6%, In 15.8%, Sn 10.3%, and Cd 2.1%, with the remainder being other rare metals, was purchased from Dongguan Metal Technology Co., Ltd., Dongguan, China.

### 2.2. Synthesis of EP and LMPA/EP Composites

**Figure 2:**
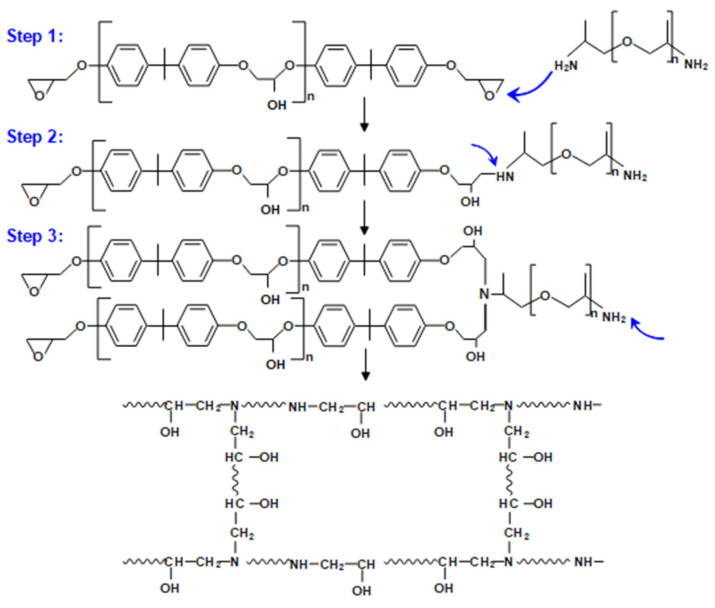
The curing reaction mechanism of D-230 curing E-51.

D-230 curing of E-51 can be divided into three steps, as shown in Figure 2. Step 1: The PA group will trigger a ring-opening reaction of epoxy resin, that is, an active hydrogen atom on PA will attack the epoxy group and generate secondary amine (SA) and a hydroxyl group. Step 2: The hydrogen atom in the SA group continues to initiate a ring-opening reaction of the epoxy group to form a tertiary amine (TA) group and a new hydroxyl group. Step 3: Polyamine further reacts with the epoxy group to gradually form a three-dimensional cross-linked network structure.

**Figure 3:**
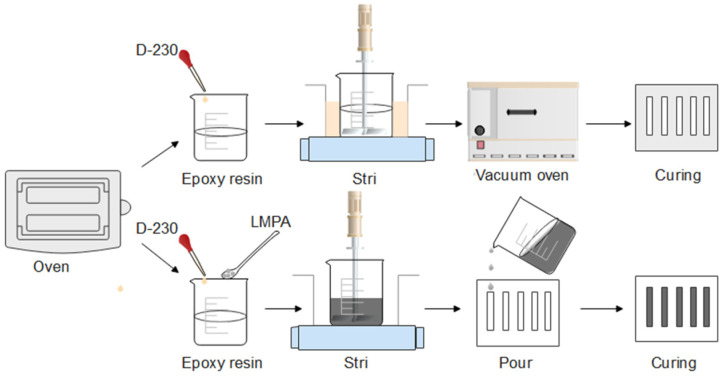
The preparation process of EP and LMPA/EP composites.

For the preparation of EP, the E-51 was placed in a beaker and heated up to 60 °C using an oven. The D-230 was then added to the beaker, and the mass ratio of E-51 to D-230 was 3:1. Then, the mixture was transferred to an oil bath at 60 °C and stirred at 300 rpm for 10 min. The vacuum oven was heated to 60 °C, and the mixture was placed in the vacuum oven for degassing for 10 min. Finally, the mixture was poured into a mold and cured, as shown in Figure 3.

For the preparation of LMPA/EP composites, E-51 12.00 g, D-230 4.00 g, and LMPA 13.8 g were blended in a beaker. Then, the beaker was placed into a 60 °C oil bath and the LMPA filler was heated to melt. Subsequently, the mixture was stirred at 300 rpm for 10 min. Finally, the mixture was poured into a preheated mold and cured at room temperature, and it was named 10 vol.% LMPA/EP. Similar operations were performed to fabricate 30 vol.% and 50 vol.% LMPA/EP, as shown in Figure 3.

### 2.3. Characterizations

#### 2.3.1. DMA Tests

The shape memory effect was tested by a dynamic mechanical analyzer (DMA, TA Instruments Q-800, New Castle, DE, USA) in the tension mode under the preset program, and the specimen was placed in the vertical direction. During the DMA test, the strain was recorded to calculate the shape fixity ratio and shape recovery ratio. The process of shape memory tests included the following five steps. First, the sample was heated at 5.0 K/min to 80 °C. Secondly, stress around 0.3 MPa was loaded and then cooled from 80 °C to 25 °C at 10.0 K/min. Thirdly, the sample was kept isothermal at 25 °C for 5 min under the load. Next, the stress was unloaded. Finally, the sample was heated back to 80 °C for the recovery of deformation. The above steps were repeated twice.

Shape fixity ratio ($R_{f}$) and shape recovery ratio ($R_{r}$) were calculated as follows:

(1) $$R_{f}=\frac{\varepsilon_{fix}}{\varepsilon_{load}}\times 100\%$$

(2) $$R_{r}=\frac{\varepsilon_{fix}-\varepsilon_{rec}(N)}{\varepsilon_{load}-\varepsilon_{rec}(N-1)}\times 100\%$$

where $\varepsilon_{load}$ is the mechanical deformation, $\varepsilon_{fix}$ is the deformation without an external force, and $\varepsilon_{rec}$ is the final deformation after *N* cycles.

The storage modulus was evaluated using a dynamic mechanical analyzer (Tritec-2000, Triton Technology, London, UK) at a single cantilever mode from 30.0 °C to 80.0 °C with a heating rate of 5.0 K/min and under the oscillation frequency of 1 Hz. The samples had a size of 30 mm × 10 mm × 2 mm.

#### 2.3.2. Split Hopkinson Pressure Bar Tests

Quasi-static compression tests were performed by using the machine (Gleeble-3180, Dynamic System Inc, AUSTIN, TX, USA) at the strain rate of 0.01/s at room temperature. The dimension of the specimen for the quasi-static compression test was 5 mm in length and 10 mm in diameter.

**Figure 4:**
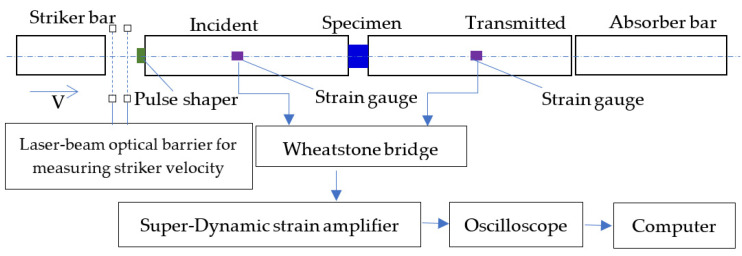
The schematic of the SHPB apparatus.

The dynamic mechanical properties were tested by a split Hopkinson pressure bar. The schematic graph of the SHPB configuration is shown in Figure 4. As shown in Figure 4, the split Hopkinson pressure bar (SHPB) consists of a striker bar, an incident bar, and a transmitted bar, and the specimen was placed between the incident and transmitted bars. Strain gauges were mounted on the middle of the incident and transmitted bar, which were used to record the incident, reflected, and transmitted stress wave. The striker bar is launched and impacts with the incident bar, and the impact induces a compression stress wave to propagate in the incident bar toward the specimen. When the stress wave reaches the interface between incident bar and specimen, an elastic tensile stress wave is reflected into the incident bar and an elastic compression wave transfers into the transmitted bar. The theory for wave propagation in bars can be used to calculate the specimen response from the signals measured by strain gauges mounted on the incident and transmitted bars. Strain gauges mounted on the incident bar were used to measure the incident ($\varepsilon_{i}$) and reflected ($\varepsilon_{r}$) strain pulses, and strain gauges mounted on the transmitted bar recorded the transmitted strain pulse ($\varepsilon_{t}$). The dynamic stress (σ), strain (ε), and strain rate ($\dot{\varepsilon }$) of the specimen can be calculated using the following formula [42]:

(3) $$\sigma =\frac{A_{b}}{A_{s}}E\varepsilon_{t},\;\varepsilon =\frac{2c}{l}\int \varepsilon_{r}dt,\dot{\varepsilon }=\frac{2c}{l}\varepsilon_{r}$$

where $A_{b}$ and $A_{s}$ are the area of the cross-section of the pressure bar and specimen, respectively; E is the elastic modulus of the pressure bar; l is the length of the specimen; c is the velocity of the stress wave in the pressure bar $c=\sqrt{E/\rho }$.

In the present paper, all bars were made of aluminum alloy with Young’s modulus E = 68 GPa and density ρ = 2780 kg/m^3^. The length of the incident and transmitted bar was 1000 mm, the length of the striker bar was 300 mm, and the diameter of all the pressure bars was 16 mm. The specimen for the SHPB was a shaft of 6 mm in length and 12 mm in diameter. The data-sampling rate was 25 MHz for all SHPB tests. In present study, the resistance strain gauge of 120 Ω (product of Zhonghang Electronic Measuring Instruments Co., LTD., Xi’an, China) was used and recorded the stress wave signals, and the strain gauges were connected by a Wheatstone half-bridge. The signals measured by gauges were amplified 1000 times by a Sinocera YE3818C super-dynamic strain amplifier (product of Sinocera piezotronics. Inc., Yangzhou, China), and recorded in a Tektronix MDO3034 oscilloscope (product of Tekronix, Beaverton, OR, USA). The frequency bandwidth of the super-dynamic strain amplifier and oscilloscope was 100 kHz and 350 MHz, respectively. A laser-beam optical barrier was used for measuring striker velocity.

**Figure 5:**
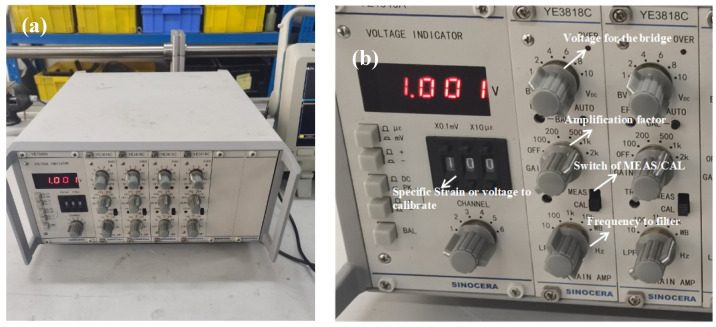
The super-dynamic strain amplifier used in the SHPB system. (**a**) graph of YE3818C; (**b**) introduction of the buttons.

The graph of the YE3818C super-dynamic strain amplifier is shown in Figure 5. As shown in Figure 5, there are two different work modes for the super-dynamic strain amplifier: (1) strain measurement mode (MEAS); (2) calibration mode (CAL). When the switch of the CAL mode is on, the calibration of the relationship between the strain and voltage is made, and the process of the strain gauge calibration is as follows: (1) a specific strain (for example, 1000 με) is sent to the bridge by the super-dynamic strain amplifier, which is also amplified by 1000 times, as shown in Figure 5; (2) then, a corresponding voltage would be obtained in the oscilloscope (for example, 0.5 V); (3) this means that 1000 με corresponds to 0.5 V, and a relationship is developed between the strain and voltage.

The principle of the SHPB experiment has two assumptions: (a) one-dimensional stress wave; (b) stress and strain in specimens are uniformly distributed along the axial direction. In the experiment, the first hypothesis was satisfied by using a pressure bar with a large aspect ratio, and the ratio of the length to diameter was 1000/16 = 62.5. In addition, a pulse shaper technique was used to ensure the second hypothesis. In the present paper, in order to ensure the equilibrium in the specimen and stability of the results, a copper pulse shaper was used to modulate the rising edge and filter the high-frequency component of the incident wave [42,43,44]. The dimension of the pulse shaper was Φ 6 mm × 0.5 mm.

**Figure 6:**
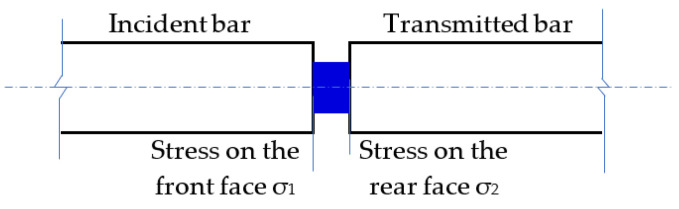
Schematic of the front and rear ends of specimens in SHPB.

As shown in Figure 6, the stresses on the front and rear faces of the specimen can be calculated according to the following formula to demonstrate the uniformity of stress in the specimen [42,43,44]:

(4) $$\sigma_{1}(t)=E[\varepsilon_{i}(t)-\varepsilon_{r}(t)]A_{0}/A_{S}$$

(5) $$\sigma_{2}(t)=E\frac{A_{0}}{A_{S}}\varepsilon_{t}(t)$$

where $\sigma_{1}\left(t\right)$ and $\sigma_{2}\left(t\right)$ are the stress on the front and rear faces of the specimen, respectively; $\varepsilon_{i}\left(t\right)$, $\varepsilon_{r}\left(t\right)$, and $\varepsilon_{t}\left(t\right)$ are the incident, reflected, and transmitted waves, respectively; $A_{s}$ is the cross-sectional area of the specimen; $A_{0}$ and *E* are the cross-sectional area and Young’s modulus of the pressure bar, respectively.

**Figure 7:**
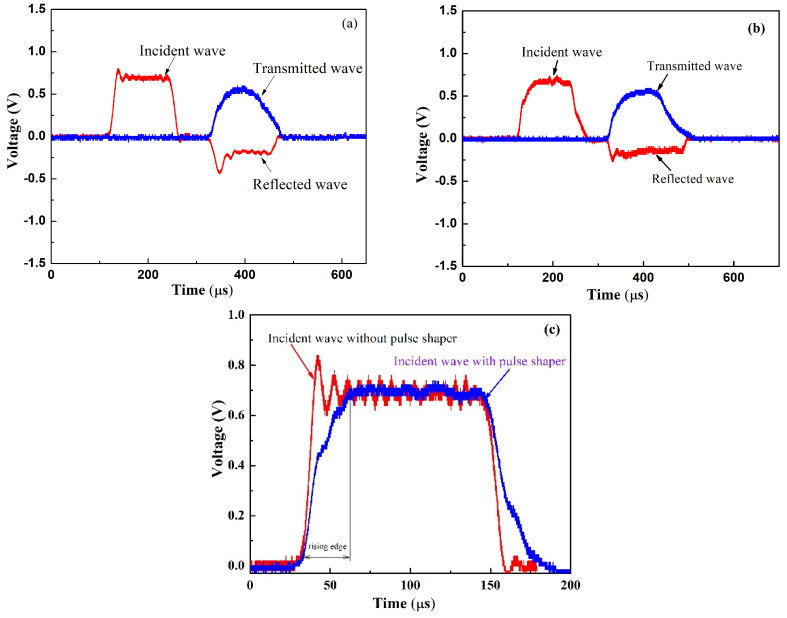
The typical stress wave in SHPB experiments. (**a**) Stress wave without a pulse shaper; (**b**) stress wave with a pulse shaper; (**c**) comparison of the different incident wave.

The typical stress waves without/with the pulse shaper are shown in Figure 7, and it can be seen from Figure 7c that the pulse shaper not only induced a rising edge of 30 μs but also filtered the high-frequency component of the stress wave, which can ensure the stress equilibrium in the specimen. Moreover, there was a disturbance in the incident wave with the pulse shaper. We considered that it was probably caused by the manufacturing defect of the pulse shaper surface, which resulted in an unstable deformation during the impact between the striker bar and incident bar. In addition, un-alignment of the bars can also result in a disturbance in the stress wave, which has been reported by Wu et al. [45].

**Figure 8:**
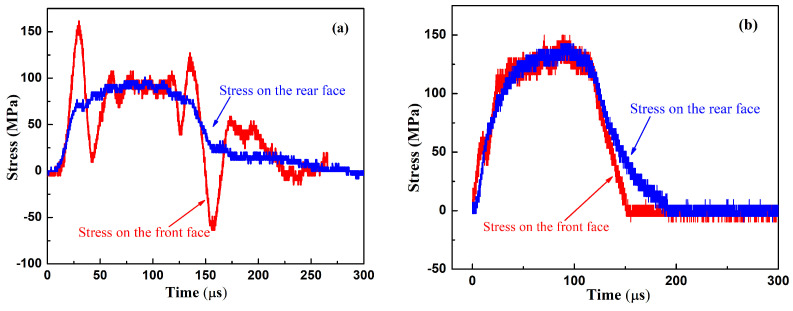
The stresses on both the front and rear surfaces: (**a**) without a pulse shaper; (**b**) with a pulse shaper.

To demonstrate the equilibrium of the stress wave in the specimen, the stresses on both the front and rear surfaces were calculated by Equations (4) and (5) and are shown in Figure 8. It can be seen from Figure 8 that the pulse shaper can improve the identity of the stresses on both the front and rear surfaces, which satisfied the hypothesis of the stress equilibrium of SHPB and ensured the reliability of the experimental results.

#### 2.3.3. SEM Tests

The distribution of LMPA in the epoxy resin matrix and the cross-section after the SHPB experiment were observed by scanning electron microscopy (Hitachi S-4800, Hitachi LTD, Tokyo, Japan) and the surfaces were sprayed with 60 s of the gold film before observation.

## 3. Results and Discussion

### 3.1. Shape Memory Effect (SME)

**Figure 9:**
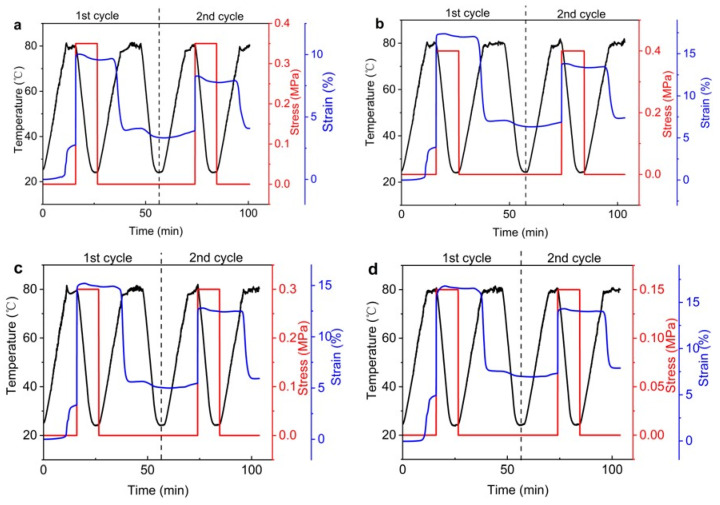
Two-dimensional variation in stress, strain, and temperature with time: (**a**) EP; (**b**) 10 vol% LMPA/EP; (**c**) 30 vol% LMPA/EP, and (**d**) 50 vol% LMPA/EP.

Figure 8 shows the results of DMA experiments at the tension mode, which gives the variations in the applied temperature, stress, and strain with time. To ensure the strains of different samples are roughly the same, different stresses were applied to the samples of different components. Figure 9a–d correspond to the results of the samples of neat EP, 10%, 30%, and 50% LMPA/EP composites, respectively. As shown in Figure 9a, the neat EP has a viscous plastic strain of 2.7% at the beginning of the first cycle, and the viscous plastic strain increases with increasing LMPA content, as shown in Figure 9b–d. The same viscous plastic strain was also observed by Yoonessi [46], which was caused by the self-gravity force of the specimen for the tensile mode of the DMA. The density of LMPA (9.6 g/cm^3^) is much larger than that of the epoxy resin (2.3 g/cm^3^); thus, the self-gravity force also increases with LMPA volume fraction, and this viscous plastic strain caused by the self-gravity force is a plastic deformation and would remain in the specimen. Thus, the viscous plastic strain in the second cycle has been subtracted and not included in the curve. In order to weaken the effect of viscous plastic deformation on SME, the strain of the second cycle was used to calculate $R_{f}$ and $R_{r}$. The mechanical deformation of neat EP in the second cycle $\varepsilon_{load}$ is 8.28%. The neat EP shows a shape recovery ratio of 96% and a shape fixity ratio of 93.7% after the second cycle.

Figure 9b–d show the stress–strain–temperature behavior for the 10%, 30%, and 50% LMPA/EP composites; the second cycle mechanical deformation is about 14%, which exceeds the deformation of the EP, and the deformation increases with increasing fraction of the LMPA. The corresponding shape recovery ratios are 95.7%, 95.5%, and 94.7%, and the shape fixity ratios are 96.6%, 97.2%, and 98.1% after the second cycle, respectively.

**Figure 10:**
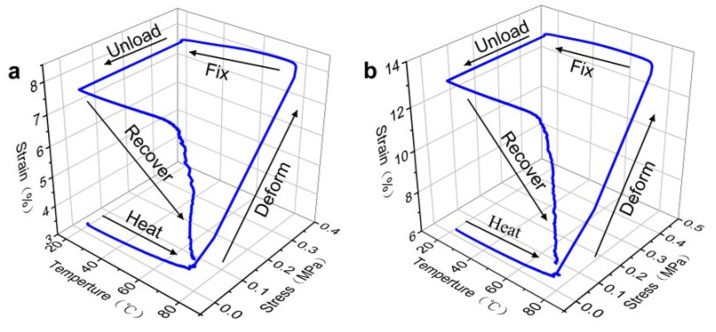
Three-dimensional SME data in the second cycle of (**a**) EP and (**b**) 10 vol% LMPA/EP.

Figure 10 illustrates the three-dimensional SME data of the second shape memory cycle of EP and 10 vol% LMPA/EP, reflecting the curves of stress–strain–temperature. It can be seen in Figure 10a,b that the shape memory cycle is divided into five stages: (1) heating to switch temperature, (2) deforming by applying a force, (3) cooling to make temporary shape fixed, (4) stress unloading, and (5) reheating to recover strain. Figure 9 illustrates each stage of the shape memory test, and it is clearly observed that the recovery of strain at the transition temperature is a rapid process.

**Figure 11:**
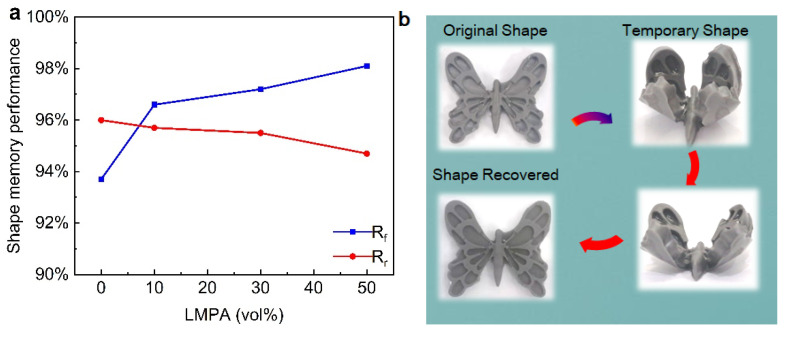
Shape memory effect: (**a**) the change in *R_f_* and *R_r_* with LMPA content; (**b**) butterfly shape restoration process.

Figure 11a illustrates the effect of LMPA content on the shape fixity ratio and the shape recovery ratio, and the corresponding parameters value is shown in Table 1. As shown in Figure 11a, it is clear that there is a slight decrease in the shape recovery ratio, but there is an obvious increase in the shape fixity ratio with increasing volume fraction of the LMPA. It is clear that LMPA does not have a contribution to the shape memory performance, but it is easy to achieve a phase exchange between solid and liquid by varying the temperature. Moreover, LMPA can also enhance the stiffness of the LMPA/EP composites. Thus, the shape fixity ratio is dominated by the LMPA additive, and the shape recovery ratio is determined by the epoxy resin volume fraction. Figure 11b shows a typical shape recovery process of a butterfly made by 30 vol.% LMPA/EP composites, which can return to its original shape in only 5 s.

**Figure 12:**
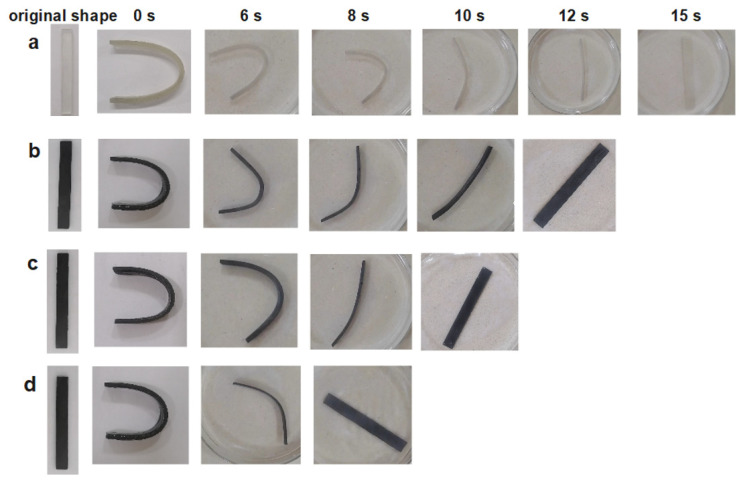
The recovery process of rectangular sample: (**a**) EP, (**b**) 10 vol% LMPA/EP, (**c**) 30 vol% LMPA/EP, and (**d**) 50 vol% LMPA/EP.

Moreover, to evaluate the accurate shape recovery time, a rectangular strip with dimensions of 80 mm × 10 mm × 2 mm was made by the LMPA/EP composites, as shown in Figure 12. It is clear that the shape recovery time is improved with increasing LMPA content. The time of the complete recovery for the EP, 10% LMPA/EP, 30% LMPA/EP, and 50% LMPA/EP is 15 s, 12 s, 10 s, and 8 s, respectively. The additive of the LMPA can improve the recovery process of LMPA/EP composites efficiently.

### 3.2. Storage Modulus

**Figure 13:**
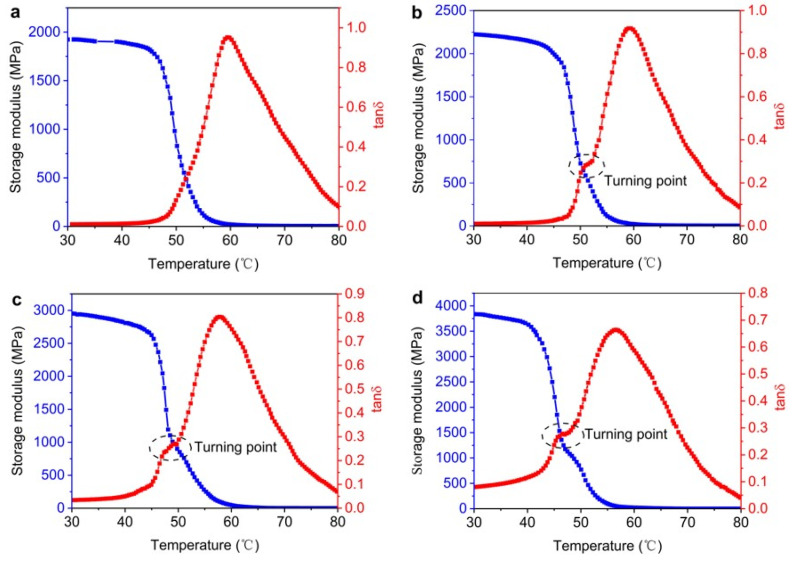
Dynamic mechanical responses of (**a**) EP, (**b**) 10 vol% LMPA/EP, (**c**) 30 vol% LMPA/EP, and (**d**) 50 vol% LMPA/EP.

Figure 13 displays the results of DMA experiments by using the single cantilever mode, which shows the ability of the storage elastic deformation and the glass transition temperatures. In addition, the loss factor Tanδ is defined as $\mathrm{Tan}\delta =E^{\prime \prime }∕E^{\prime }$, and $E^{\prime \prime }$ and $E^{\prime }$ are the loss modulus and storage modulus, respectively. The temperature corresponding to the peak of the loss factor is defined as the glass transition temperature *T_g_* [47]. As shown in Figure 13a, the modulus of EP decreases sharply by 3 orders of magnitude as the temperature increases from 30 °C to 80 °C, which represents the samples having excellent shape memory effects, and the *T_g_* of EP is around 59.4 °C. It can be seen that there is an obvious turning point in loss factor curves in Figure 13b–d, and the region value of the turning point ΔT is 1.6 °C (50.6–52.2 °C), 1.9 °C (47.8–49.7 °C), and 2.2 °C (45.5–47.7 °C) for the 10% LMPA/EP, 30% LMPA/EP, and 50% LMPA/EP composites, respectively. It can be concluded that the start temperature of the turning point decreases with increasing LMPA content (from 50.6 °C to 45.5 °C); however, the region value increases with the increase in the LMPA volume fraction (from 1.6 °C to 2.2 °C). For the region value of the turning point: the strength of the LMPA is much lower than the EP. The melting of LMPA would decrease the modulus of the LMPA/EP composites and result in the loss modulus. Moreover, the larger the LMPA content, the greater the requirement of the heat. For the start temperature of the turning point: though the melting point of LMPA is about 46 ± 0.5 °C, the LMPA in the 10% LMPA/EP composites would not melt completely when the temperature is heated to 46 °C. The reason is the LMPA is distributed in the EP matrix and the LMPA content of 10% is too little, and the heat cannot transfer from the EP matrix to all of the LMPA completely, which needs a slightly higher temperature to achieve the transferring process in 10% LMPA/EP composites. However, for the increasing LMPA content, such as 50% LMPA/EP, the distribution of LMPA in the EP matrix is much larger, and it is much easier to achieve heat transformation from EP to LMPA. Thus, the corresponding temperature of the turning point decreases with increasing LMPA content. Moreover, when the LMPA volume content is up to 50%, the glass transition temperature *T_g_* decreases by 3 °C in contrast to neat EP samples. The result indicates that the LMPA has little effect on the *T_g_*.

**Figure 14:**
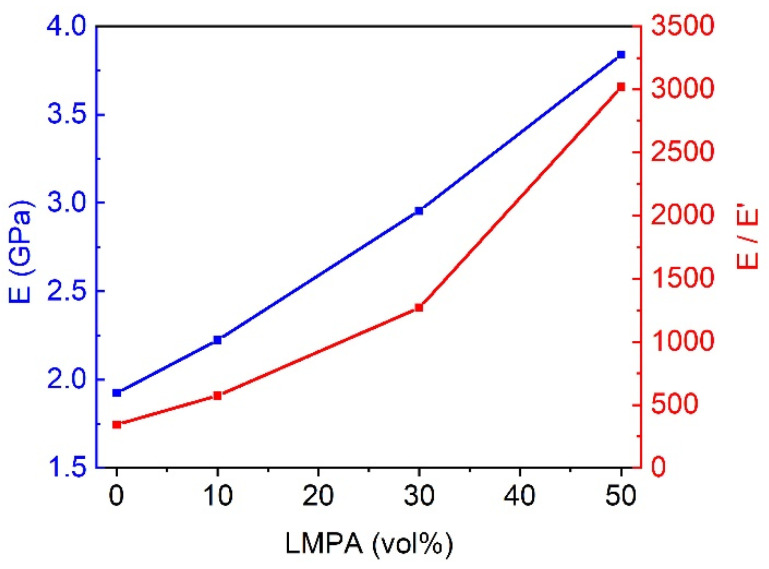
Storage modulus *E* of sample at 30 °C and the ratio of the modulus at 30 °C to 80 °C (*E/E*’).

Figure 14 shows the influence of LMPA content on the modulus of the glassy state (30 °C) and the ratio of the modulus of the glassy state to rubber state (80 °C). As shown in Figure 14, for the temperature of 30 °C (*T* > *T_g_*), the storage modulus (*E*) of composites grows linearly with augmented LMPA content, and the storage modulus (*E*) changes from 1.92 GPa to 3.84 GPa, which demonstrates that the LMPA has a significant contribution to the enhancement of the storage modulus. For the temperature of 80 °C (*T* > *T_g_*), the storage modulus (*E*’) of composites decreases slightly. It is clear that the storage modulus ratio (*E/E*’) varies in the range from ≈400 to over ≈3000. Thus, the addition of LMPA amplifies the storage modulus of the glass state and reduces the modulus of the rubber state, which means that the tunable stiffness of the composites is expanded.

**Figure 15:**
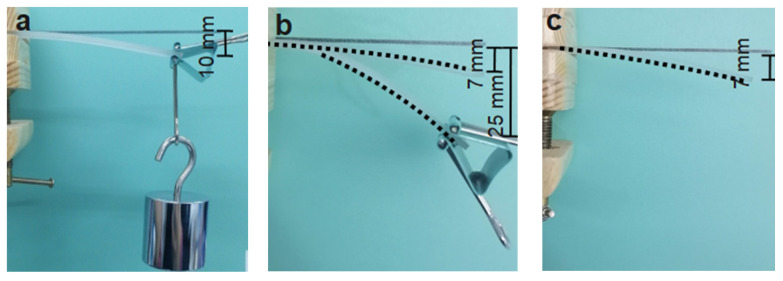

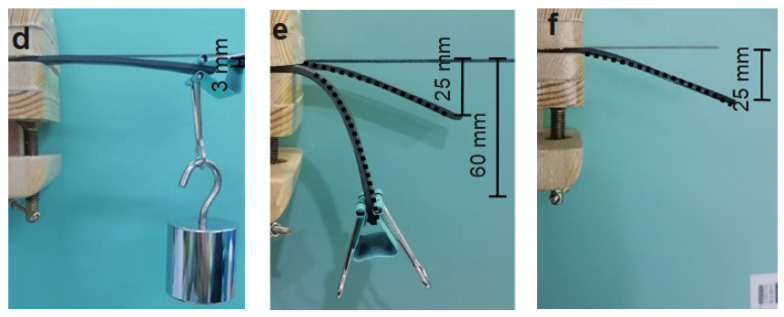
The left and middle columns are the changes in stiffness of EP (**a**,**b**) and 30 vol% LMPA/EP (**d**,**e**) at room temperature and 80 °C. The specimen is in a high elastic state at high temperature, and the clamp is used as the load alone (middle column). The right column represents the shape recovery after heating with the load removed (**c**,**f**).

In order to demonstrate the variable stiffness influenced by the LMPA, the deflection deformation test of the cantilever beam was performed, and the beam was made by the EP and 30 vol% LMPA/EP. The size of the beam is 80 mm × 10 mm × 2 mm, as shown in Figure 15. At room temperature, the deflection of neat EP under a load of 200 g is 10 mm, which is larger than the 3 mm deflection of the LMPA/EP beam (Figure 15a,d). The deflection test also verifies that the stiffness of the LMPA/EP beam is larger than that of neat EP, which means the addition of LMPA increases the stiffness. However, when the ambient temperature is heated up to 80 °C, the deflection of the LMPA/EP beam is 25 mm under the self-weight loading, which is much larger than that of the EP beam (7 mm), which means that the stiffness of LMPA/EP decreases at high temperature (as shown in Figure 15b,e). If a clamper as a load were to act on the beam, the deflection of the LMPA/EP beam would increase to 60 mm. The larger the deflection, the softer the material, which also means the less rigid the material. Deflection experiments also confirmed that the stiffness of the LMPA/EP composite is higher than that of the neat EP at room temperature and much lower than that of neat EP above the transition temperature. Finally, the beam was cooled to room temperature and the load of the clamper was removed, meaning that the beam can return to the position at 80 °C (as shown in Figure 15c,f).

### 3.3. Dynamic Mechanical Response at Different Strain Rates

#### 3.3.1. Stress–Strain Curves at Various Strain Rates

**Figure 16:**
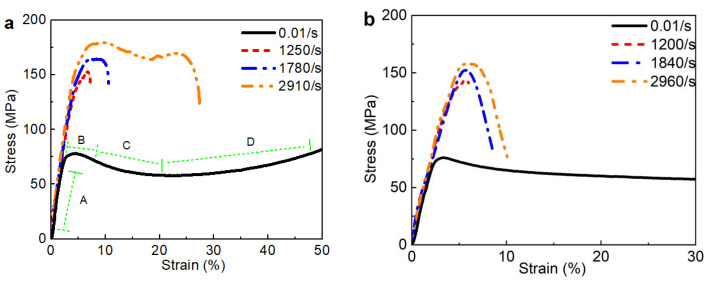

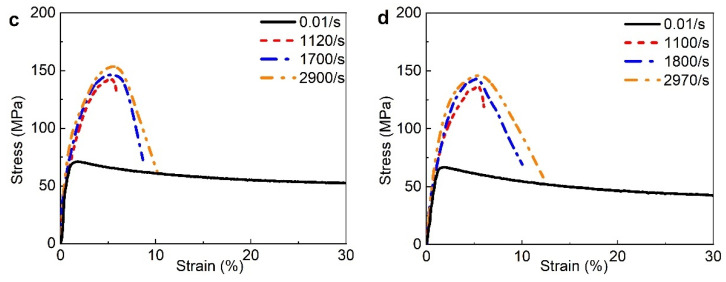
Stress–strain curves of composites at various strain rates. (**a**) EP; (**b**) 10 vol% LMPA/EP; (**c**) 30 vol% LMPA/EP; (**d**) 50 vol% LMPA/EP.

Figure 16 illustrates the stress–strain curves of the LMPA/EP composites at various strain rates. Figure 16a–d correspond to the stress–strain curves of the EP, 10%, 30%, and 50% LMPA/EP. It is clear that there is a significant difference in the flow stress between the quasi-static and high-strain-rate load cases, and the flow stress increases obviously at high strain rates compared to the quasi-static load case. Generally, the peak stress under quasi-static load is about 70 MPa; however, the peak stress at high strain rate is about 140~170 MPa, which indicates that the flow stress of the LMPA/EP composites is sensitive to the strain rate. In addition, the flow stress at high strain rate also increases with strain rate, but such an increasing tendency is slow with the increase in the strain rate.

Under the quasi-static compression, the stress–strain curves of EP and LMPA/EP composites exhibit the typical ductile characteristics, which includes four stages: elastic stage (A), yield stage (B), strain soften stage (C), and stain harden stage (D), as shown in Figure 16a. However, the shape of the stress–strain curve at high strain rate is completely different from that under quasi-static loading. Especially, as shown in Figure 16b–d, the strain at high strain rate is only about 10%, and much less than the strain (about 30%) under quasi-static loading, which indicates that the deformation ability decreases seriously at high strain rate.

**Figure 17:**
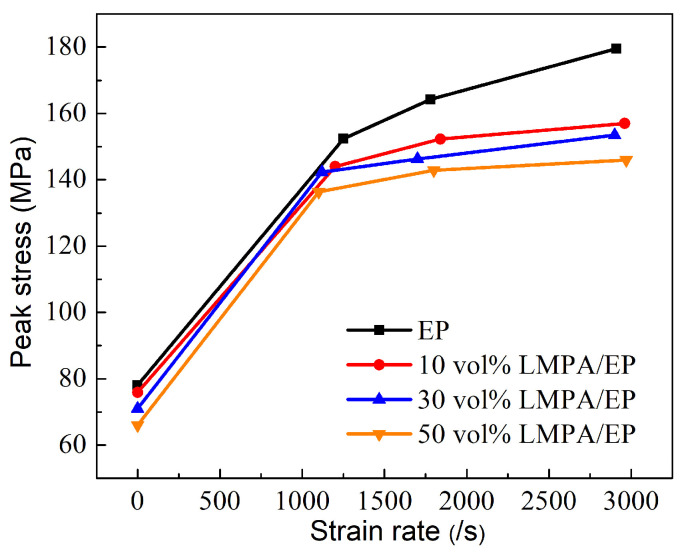
The variations in the peak stress with strain rate.

The variations in the peak stress of the composites with the strain rate are shown in Figure 17. It can be observed from Figure 16 that the peak stress of the composites increases with the increasing strain rate, but the increasing amplitude decreases with the increase in the LMPA content, which is due to the heat softening converted by the plastic work at high strain rates. The heat generated by plastic work can be calculated by α∫σdε, and α is the coefficient of the heat generated by plastic work. Similar works have been reported by Tan [44] and Arruda [48]. The melting point of the LMPA is about 47 °C, and the local temperature rise in the composites would be larger than this melting point of 47 °C [48]. When the LMPA is in melting status, it would not suffer a load anymore. Thus, the maximum of the stress would reach a saturated status. Moreover, the peak stress of the LMPA/EP composites decreases with increasing LMPA volume fraction. For such LMPA/EP composites, it can be considered as a foam EP matrix filled with LMPA. With the LMPA content increasing, the foam EP matrix would be high porosity, which has a low strength. Thus, the peak stress of the LMPA/EP composites decreases with increasing volume fraction of the LMPA.

#### 3.3.2. Macroscopic Failure Modes and SEM Characterization

**Figure 18:**
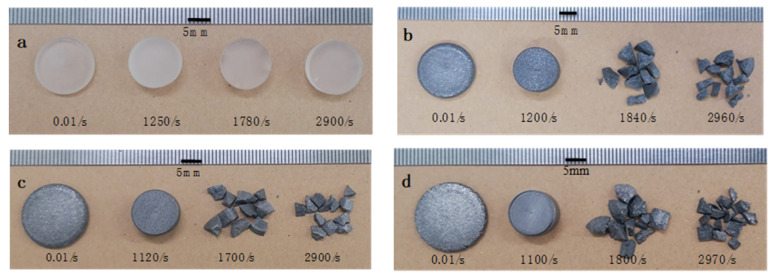
The failure modes of the specimens at various strain rates: (**a**) EP; (**b**) 10 vol% LMPA/EP; (**c**) 30 vol% LMPA/EP; (**d**) 50 vol% LMPA/EP.

Figure 18 shows the failure modes of the tested samples at various strain rates. It is clear that the additive of LMPA has an obvious influence on the failure mode of the LMPA/EP composites compared to the neat EP. Both EP and LMPA/EP composites exhibit an excellent plastic deformation under the quasi-static compression, and the neat EP can still maintain the ductile deformation with some cracks at high strain rates. However, for the LMPA/EP composites, the fracture failure modes occur at high strain rates, which shows the typical brittle properties.

**Figure 19:**
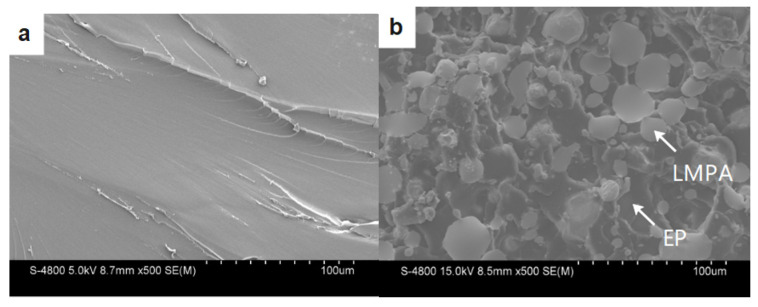

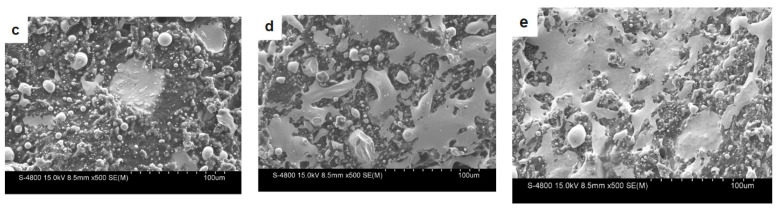
SEM images before SHPB tests of (**a**) EP and (**b**) 50 vol% LMPA/EP; the SEM images of fracture surface of the specimens after SHPB tests at the strain rate of about 1800 s^−1^: (**c**) 10 vol% LMPA/EP, (**d**) 30 vol% LMPA/EP, and (**e**) 50 vol% LMPA/EP.

Based on the macro-failure modes in Figure 18, in order to explain the deformation mechanism more clearly, the SEM tests were performed on the specimen before and after SHPB tests, as shown in Figure 18. For the specimen before the SHPB tests, Figure 19a,b show the SEM graphs of the typical microstructure of the EP and PCL/EP composites. The SEM graph of the EP shows a homogeneous distribution, and there is no pore or cavity defect in the EP, as shown in Figure 19a. Figure 19b shows the dispersion of LMPA in the matrix; the spherical particles are LMPA. It is obvious that the LMPA is distributed in the epoxy resin matrix uniformly, which can ensure the reliable performance of the LMPA/EP composites.

For the specimen after the SHPB test, the SEM tests were performed on the fracture surface of the tested samples. Figure 19c–e show the SEM image of the fracture surface of the composites sample. It is clearly observed that there is some molten LMPA on the fracture surface of 10 vol.% LMPA/EP composites in Figure 19c. Moreover, the melting phenomenon is more obvious on the fracture surface of 30 vol.% and 50 vol.% LMPA/EP in Figure 18d,e.

### 3.4. Discussions

#### 3.4.1. The Influence of the LMPA on the SME of LMPA/EP Composites

It can be seen from Figure 10, Figure 11 and Figure 12 that LMPA/EP composites exhibit excellent shape memory performance. Due to the LMPA material not having the shape memory ability, the shape memory performance of LMPA/EP is dominated by the epoxy resin matrix. The shape memory process includes: (1) When the temperature is higher than the LMPA melting point $\left(T_{d}\right)$, the LMPA material starts to melt and be in a liquid state. (2) When the temperature is above $T_{g}$, molecular chains of epoxy resin begin to move, and the epoxy resin transforms into a rubbery state. (3) At this point, an external force is applied to induce the deformation of the sample. As the temperature drops below $T_{d}$, the LMPA would return to the solid state, and the epoxy resin transforms into a glassy state, so a temporary shape is obtained. (4) When the temperature is heated above the glass transition temperature of the EP, the internal stress stored by the molecular chain is released, and the shape returns to the original shape. Hence, the mechanism can be concluded as follows: the shape memory performance of LMPA/EP composites is dominated by epoxy resin matrix, and LMPA has a significant contribution to improve the shape fixation rate of LMPA/EP composites.

#### 3.4.2. The Influence of the LMPA on the Failure Mechanism of LMPA/EP Composites

**Figure 20:**
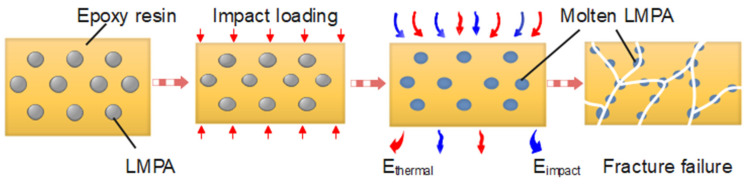
Schematic graph of the failure mechanism of LMPA/EP composites.

In the following work, the deformation mechanism of the LMPA/EP composites at high strain rate is discussed, and the corresponding schematic graph is also shown in Figure 20. Under quasi-static compression, the molecular chains in the epoxy resin matrix have sufficient time orientation, which ensures the excellent plastic deformation; under dynamic compression, the duration of the impact load pulse is very short (~200 μs), and the heat generated by the plastic work cannot be diffused, which can be considered as adiabatic compression, which would make the LMPA molten. In addition, the molten LMPA is in the liquid phase, which cannot bear the load anymore, and the external load would be burdened by the epoxy resin matrix. Based on the SEM graph of LMPA/EP composites in Figure 19b, it can be observed that the epoxy resin matrix is filled with the LMPA particles. Thus, the epoxy resin matrix can be considered as a porous structure material, and the LMPA fills these holes in the epoxy resin matrix. When the LMPA is molten by the heat converted by the plastic work at high strain rate, the porous epoxy resin matrix would bear the impact loading. Finally, the hole wall of the porous structure fails, and the molten LMPA covers the fracture surface of the epoxy resin matrix, which corresponds to the fracture failure mode and molten phenomena of the LMPA in Figure 18 and Figure 19c–e. It also indicates that the LMPA/EP composites are more suitable for low-strain-rate service environments due to its excellent plastic deformation under quasi-static compression compared to the dynamic load case.

## 4. Conclusions

In the present paper, LMPA was used to fabricate the epoxy-resin-based SMP composites, and different DMA experiments were conducted to demonstrate that the LMPA has an obvious influence on the stiffness, shape fixity rate, and shape memory effect, which can modulate the stiffness effectively. Moreover, the dynamic mechanical properties of the SMP composites were studied by SHPB experiments at various strain rates, where there was an obvious difference in flow stress and failure mode mechanism between the quasi-static and dynamic load case. The flow stresses at high strain rates were much larger than that at quasi-static loading. For the composites with different LMPA content, the peak stress at high strain rate was nearly two times the peak stress under quasi-static compression loading. Moreover, the LMPA/EP composites showed an excellent plastic deformation ability under quasi-static compression; however, the composites exhibited typical brittle failure modes at high strain rates, and the melting phenomena of the LMPA were observed on the fracture surface of the tested samples by SEM tests, which was attributed to the heat generated from the plastic deformation during the dynamic compression. Based on the above results, the deformation and failure mechanisms were also discussed.

For the perspective applications, although LMPA/EP composites failed at a high strain rate, the composites can sustain a quasi-static load in a normal service environment such as medical fixed equipment, robot control, and folding structures. For some emergency cases, such as an accident in the folding and locking structure, LMPA/EP composites can be failed by exerting an impact loading, which can solve the accident quickly and efficiently. Moreover, the LMPA/EP composites can absorb the energy through plastic deformation and the solid–liquid phase transformation process, which can be used to protect the electric device in vehicle and aerospace equipment. In the future, we will focus on the optimal design of the LMPA/EP composites to improve the energy absorption and deceleration performance.

## Figures and Tables

**Table 1:** The parameters for the shape fixity ratio and the shape recovery ratio.

Sample	*ε_rec_*(1)	*ε_rec_*(2)	*ε_load_*	*ε_fix_*	*R_f_*	*R_r_*
EP	3.93	4.08	8.28	7.76	93.7%	96%
10 vol% LMPA/EP	6.99	7.28	13.82	13.35	96.6%	95.7%
30 vol% LMPA/EP	5.61	5.93	12.80	12.44	97.2%	95.5%
50 vol% LMPA/EP	7.54	7.90	14.31	14.04	98.1%	94.7%

## Data Availability

The raw data needed to reproduce these findings cannot be shared at this time, as the data will be used in ongoing research.
